# Human proximal tubular cells can form calcium phosphate deposits in osteogenic culture: role of cell death and osteoblast-like transdifferentiation

**DOI:** 10.1038/s41420-019-0138-x

**Published:** 2019-01-28

**Authors:** Giovanna Priante, Monica Ceol, Lisa Gianesello, Claudio Furlan, Dorella Del Prete, Franca Anglani

**Affiliations:** 1 Laboratory of Kidney Histomorphology and Molecular Biology, Clinical Nephrology, Department of Medicine-DIMED, University of Padova, Padova, Italy; 2 Center for ESEM and SEM analyses (CEASC), University of Padova, Padova, Italy

## Abstract

Nephrocalcinosis is a clinicopathological entity characterized by microscopic calcium crystals in the renal parenchyma, within the tubular lumen or in the interstitium. Crystal binding to tubular cells may be the cause underlying nephrocalcinosis and nephrolithiasis. Pathological circumstances, such as acute cortical necrosis, may induce healthy cells to acquire a crystal-binding phenotype. The present study aimed to investigate whether human renal proximal tubular cells (HK-2 cells) can form calcium phosphate deposits under osteogenic conditions, and whether apoptosis and/or osteogenic-like processes are involved in cell calcification. HK-2 cells were cultured in standard or osteogenic medium for 1, 5, and 15 days. Von Kossa staining and ESEM were used to analyze crystal deposition. Apoptosis was investigated, analyzing caspase activation by in-cell Western assay, membrane translocation of phosphotidylserine by annexin V-FITC/propidium iodide staining, and DNA fragmentation by TUNEL assay. qRT/PCR, immunolabeling and cytochemistry were performed to assess osteogenic activation (Runx2, Osteonectin, Osteopontin and ALP), and early genes of apoptosis (BAX, Bcl-2). HK-2 cell mineralization was successfully induced on adding osteogenic medium. Calcium phosphate deposition increased in a time-dependent manner, and calcified cell aggregates exhibited characteristic signs of apoptosis. At 15 days, calcifying HK-2 cells revealed osteogenic markers, such as Runx2, ALP, osteonectin and osteopontin. Monitoring the processes at 1, 5, and 15 days showed apoptosis starting already after 5 days of osteogenic induction, when the first small calcium phosphate crystals began to appear on areas where cell aggregates were in apoptotic conditions. The cell death process proved caspase-dependent. The importance of apoptosis was reinforced by the time-dependent increase in BAX expression, starting from day 1. These findings strongly support the hypothesis that apoptosis triggered HK-2 calcification even before any calcium phosphate crystal deposition or acquisition of an osteogenic phenotype.

## Introduction

Nephrocalcinosis is a clinicopathological entity characterized by microscopic calcium crystal (calcium oxalate or calcium phosphate) deposition in the renal parenchyma, either within the tubular lumen (intratubular nephrocalcinosis) or in the interstitium (interstitial nephrocalcinosis). Nephrocalcinosis can be classified as medullary or cortical. Medullary nephrocalcinosis is the typical pattern (seen in 98% of cases of human nephrocalcinosis), with calcification clustering around each renal pyramid. It is common in patients with metabolic conditions that predispose them to calcium renal stones^1–4^. Cortical nephrocalcinosis is rare, and usually due to severe cortex destruction^5–10^ due to any condition causing acute and prolonged shock^10–12^.The characteristic cortical calcification develops within a few weeks. The medullary pyramids are usually spared, retaining soft tissue attenuation. When cortical nephrocalcinosis first appears, the kidneys are still enlarged due to inflammatory edema, but with time they become atrophic.

Ectopic calcification is known to follow necrosis, and cortical nephrocalcinosis has been attributed to the presence of necrotic tubular cells^13,14^. To our knowledge, the role of cell death in the more common medullary nephrocalcinosis remains unclear. The most accredited explanation for the onset of nephrocalcinosis is purely physicochemical, involving spontaneous calcium phosphate crystallization in the tubuli or in the interstitium due to its oversaturation with calcium phosphate salts^14,15^. Nobody knows exactly how the tubulo-interstitial cells respond to the influx of these potentially precipitating ions. Ectopic renal calcification might be an osteogenic-like process, and evidence in the literature supports the notion that resident renal cells could be prompted to transdifferentiate, or differentiate along an osteogenic lineage^16–23^. We were the first to suggest that nephrocalcinosis might be an osteogenic-like, cell-driven process, with human renal cells undergoing calcification under certain circumstances in much the same way as in vascular calcification^24–27^.

Vascular calcification was long thought to result from passive degeneration^28^, but actually involves a complex, regulated process of biomineralization similar to osteogenesis, which mediates bone matrix deposition in the blood vessels^29–40^.

The present study aimed to investigate whether HK-2 cells (a human renal proximal tubular cell line) can form calcium phosphate deposits under osteogenic conditions, and whether apoptosis and an osteogenic-like process are involved in the cell calcification process.

## Results

### In osteogenic medium, HK-2 cells form cell aggregates containing calcium phosphate

HK-2 cells were treated with osteogenic medium for 1, 5, and 15 days, and calcium phosphate deposition was monitored by von Kossa staining and ESEM analysis.

**Fig. 1:**
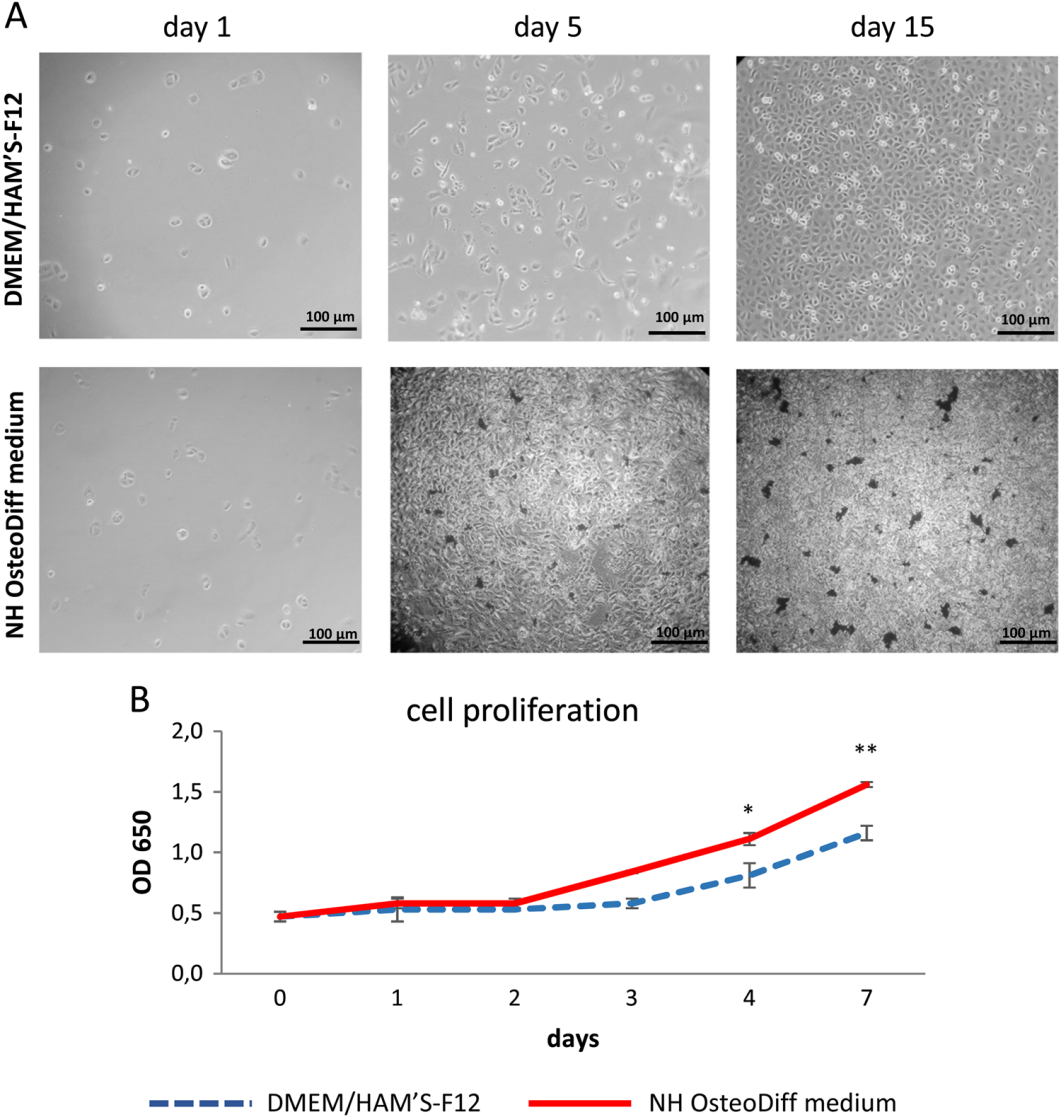
HK-2 cells grown in osteogenic medium exhibit multilayer growth with multicellular aggregates or nodules displaying dense deposits. **a** Phase-contrast inverted microscope images of cells grown in DMEM/HAM’S-F12 supplemented with 10% HI-FBS or NH OsteoDiff medium. The images are representative of three separate experiments. Bar = 100 μm. **b** Proliferation curve of HK-2 cells grown in standard and osteogenic conditions. Data are presented as mean ± SD of three separate experiments. *p < 0.05 cell number on day 4 in NH OsteoDiff medium vs cell number on day 4 in DMEM/HAM’S-F12, **p < 0.001 cell number on day 7 in NH OsteoDiff medium vs cell number on day 7 in DMEM/HAM’S-F12

In standard conditions HK-2 cells grew continuously and homogeneously as a monolayer. At 15 days, the cultures became highly confluent, with polygonal, round, and ellipsoidal cells exhibiting a characteristic cobblestone appearance (Fig. 1a). Cells grown in osteogenic medium were multilayered, retracting from some areas, and forming multicellular aggregates or nodules with dense deposits becoming evident after 5 days (Fig. 1a). This different cell growth was confirmed by analyzing cell proliferation. Monitoring from days 1 to 7 showed a similar, gradually increasing cell growth in both standard and osteogenic media (Fig. 1b). The two growth curves only overlapped on days 1 and 2, however, then cell proliferation was slower in the standard medium than in the osteogenic medium, reaching a significant maximum difference on day 7 (*p* < 0.001, Fig. 1b).

**Fig. 2:**
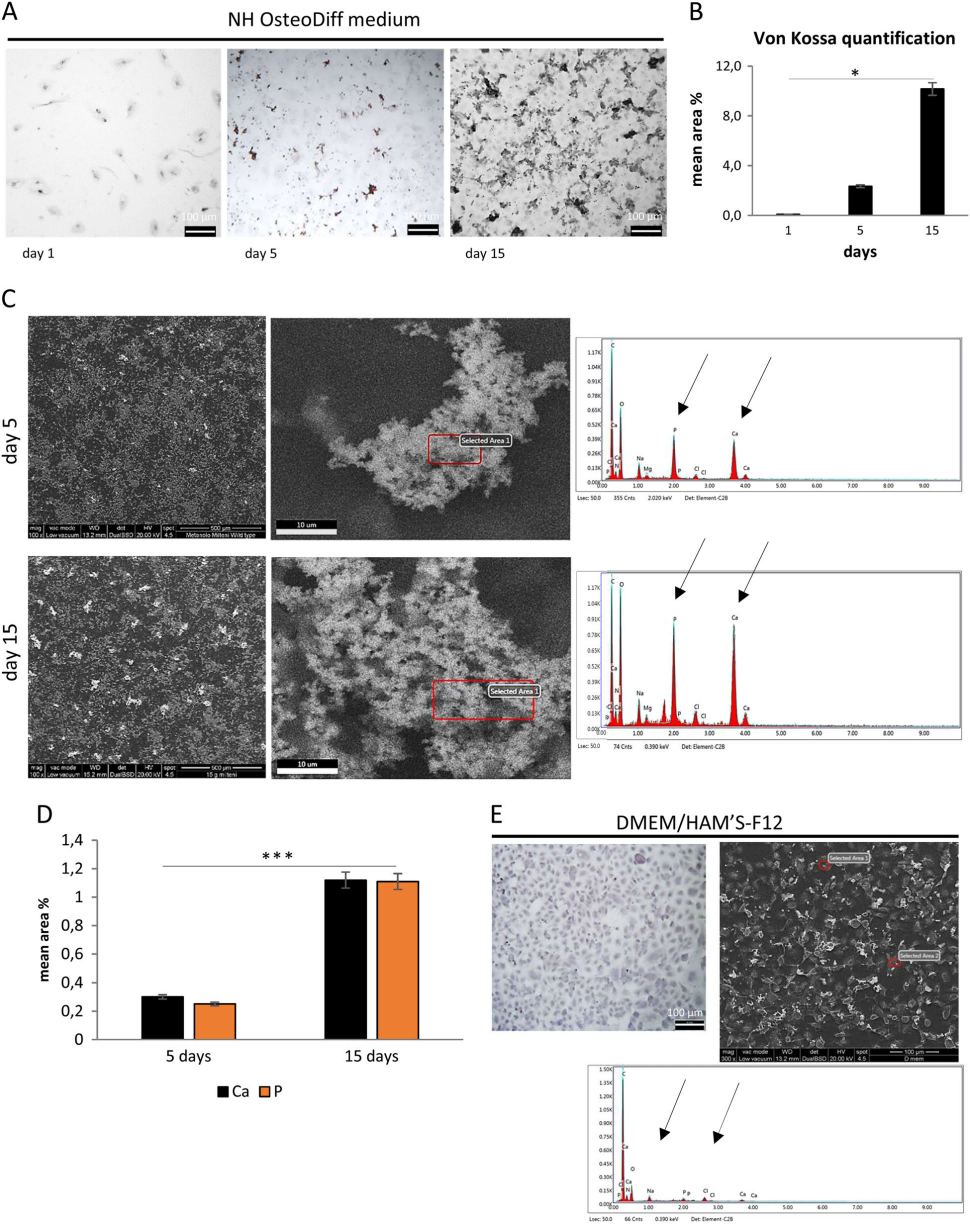
Calcium phosphate deposition is detected in HK-2 cell aggregates in osteogenic medium. **a** von Kossa staining images of cells cultured in NH OsteoDiff medium. Images are representative of three separate experiments. **b** Quantitative analysis of von Kossa staining performed using morphometric analysis on the pooled results of three separate experiments, and the values represent the means ± SD. *p < 0.05. **c** ESEM images and spectra of selected areas of cells cultured in NH OsteoDiff medium for 5 and 15 days. The images are representative of three separate experiments. Bar = 500 μm left and 10 μm right. **d** Semi-quantitative measure of the composition of the inclusions, based on three separate experiments, the values representing the means ± SD. ***p < 0.005. Ca = Calcium, P = Phosphate. **e** von Kossa staining (left) and ESEM image with representative spectrum of selected areas (right) of cells cultured. DMEM/HAM’S-F12 supplemented with 10% HI-FBS (day 15)

Von Kossa staining revealed dense deposits of calcium aggregates (Fig. 2a) in the HK-2 cells cultured under osteogenic conditions at 5 and 15 days, and morphometric analysis showed a time-dependent trend, with significantly more calcium deposition at 15 days (*p* < 0.005) than at 5 days (Fig. 2b). ESEM analysis showed that these granular concretions contained abundant Calcium (Ca) and Phosphate (P), and ranged from 1.0 to 30 μm in diameter at 5 days, and from 3.0 to 60 μm at 15 days (Fig. 2c). The quantity of Ca and P was also significantly greater at 15 days (*p* < 0.005) than at 5 days (Fig. 2d). The concomitant presence of Ca and P suggests calcium phosphate precipitation into multicellular aggregates or nodules. ESEM confirmed that no Ca and P deposition occurred in cells under standard conditions (Fig. 2e).

### Cell death in the calcified nodules of HK-2 cells

#### Induction of apoptosis: cellular and nuclear morphological analysis

**Fig. 3:**
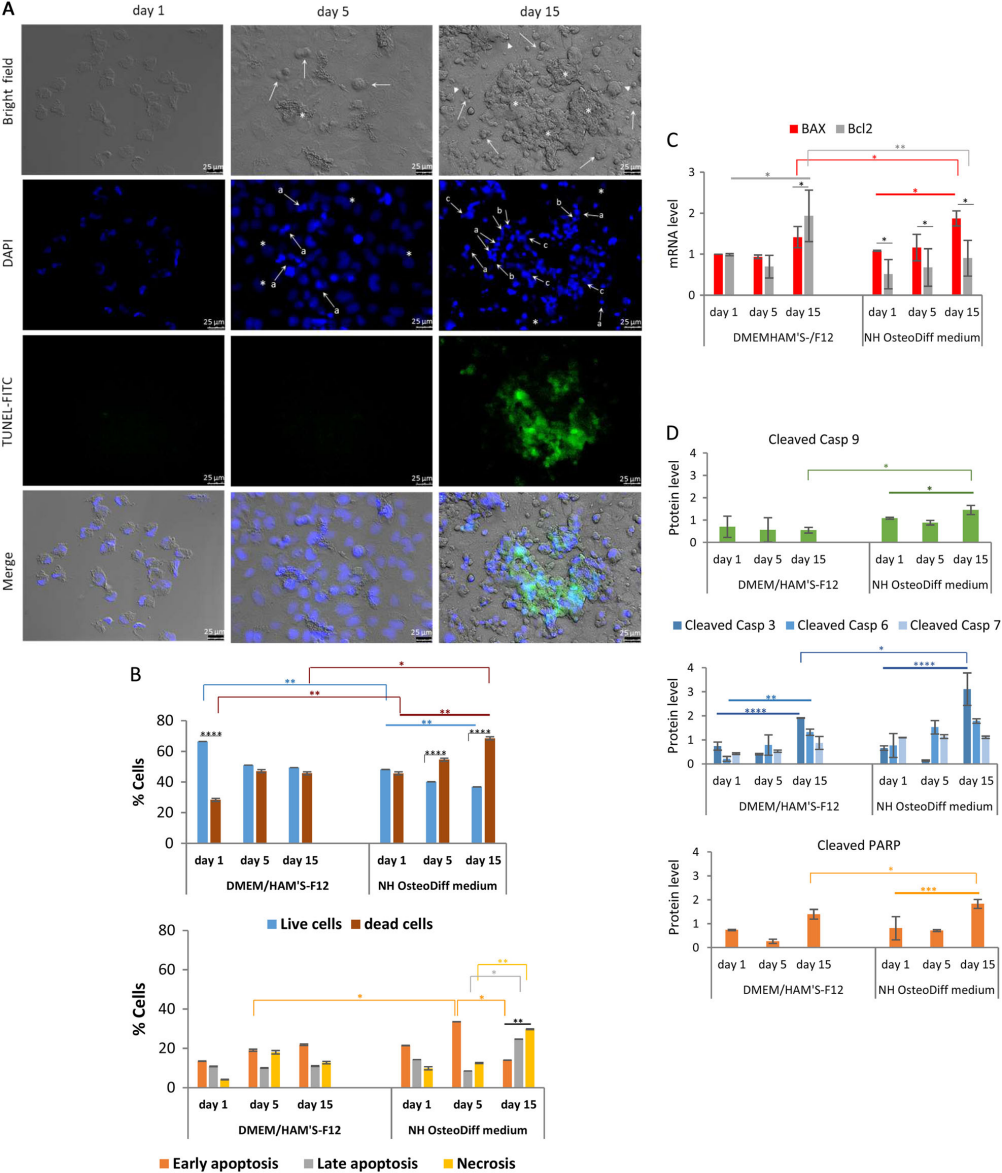
HK-2 cells in osteogenic medium show caspase-dependent apoptosis localized in cellular aggregates. **a** Representative bright field and immunofluorescence images of DAPI and TUNEL-stained apoptotic nuclei of HK-2 cells grown in NH OsteoDiff medium for 1, 5, and 15 days. In the bright field image, arrows indicate small, highly refringent circular cells with condensed cytoplasm, asterisks indicate cell aggregates or nodules, and arrowheads show examples of membrane blebbing. In the DAPI images, apoptotic nuclei are brightly blue fluoresced by comparison with normal nuclei (asterisks). Arrows point to examples of: **a** apoptotic bodies of nuclear condensation; **b** chromatin marginalization; and **c** fragmentation. FITC-TUNEL images show DNA fragmentation localized in the nodule (Merge). The images are representative of three experiments. Bar = 25 μm. **b** Quantification of dead cells by double staining of HK-2 cells with annexin V and PI, for 1, 5 and 15 days: live versus dead cells (up); and early or late apoptotic and necrotic cells (down). Dead cells = early + late apoptotic cells + necrotic cells. Data are presented as the mean ± SD of three separate experiments. **p < 0.001; ***p < 0.005; ****p < 0.0001. **c** qRT/PCR of *BAX* and *BCL2* apoptosis-related genes, for 1, 5, and 15 days. Data are presented as the mean ± SD of three separate experiments. *p < 0.05; **p < 0.001. **d** Quantification of initiator caspase-9, effector caspases -3, -6 and -7, and PARP substrate by in-cell Western assay, for 1, 5, and 15 days. Data are presented as the mean ± SD of three separate experiments. *p < 0.05; **p < 0.001; ***p < 0.005; ****p < 0.0001

Changes in cell morphology can reflect the degree of cell damage, so the cells cultured in standard and osteogenic conditions underwent morphological analysis: the former cells as expected were of normal shape, with rounded nuclei homogeneously staining with DAPI; the latter showed changes in the cytoplasmic morphology such as cell shrinkage and cell connection disappearance (Fig. 3a). Cell shedding was already apparent after 5 days, and peaked at 15 days, by which time the cells were small and highly refringent, acquiring a circular shape. There were numerous clumped cells with a condensed cytoplasm forming aggregates or nodules, where the Ca and P deposits were found. At this stage, the cells were still nearly all adherent, and also showed membrane blebbing (Fig. 3a, bright field). These morphological changes revealed the induction of apoptosis, which was further examined using nuclear morphology. Internucleosomal DNA condensation and fragmentation are important biochemical hallmarks of early and late apoptosis, respectively, representing a point of no return from the path to cell death^41^. DAPI-stained and TUNEL-labeled HK-2 cells grown in osteogenic medium revealed typical apoptotic features, such as brightly fluorescing nuclei, chromatin condensation and marginalization, apoptotic bodies, and DNA fragmentation (Fig. 3a, DAPI). While there were few positive DAPI-stained cells with apoptotic nuclear changes and no TUNEL-positive cells at 5 days, both had increased by day 15 (Fig. 3a, DAPI and TUNEL-FITC), indicating the DNA fragmentation typical of the late stage of apoptosis^42^. Intriguingly, all these DAPI-staining or TUNEL-positive cells were near or inside nodules (Fig. 3a, Merge).

#### Apoptosis vs necrosis

Apoptosis and necrosis may share several features. Double staining with annexin V-FITC and PI enabled us to discriminate between apoptotic and necrotic cell death (Fig. 3b). Under standard conditions, at 1 day, there was a higher proportion of live than dead cells (*p* < 0.0001), whereas at 5 and 15 days the number of live and dead cells was quite similar. This last picture was seen at 1 day in osteogenic conditions when the number of live and dead cells, however, was significantly lower (*p* < 0.001) and higher (*p* < 0.001) respectively than under standard conditions. Under osteogenic conditions, there was a higher overall cell death rate over the time (*p* < 0.001), and compared with HK-2 cells grown in standard conditions at 15 days (*p* < 0.05) (Fig. 3b, up). Once established that in osteogenic conditions the rate of cell death was significantly higher than in standard conditions, we focused on the various phases of cell death process, i.e., early or late apoptosis and necrosis (Fig. 3b, down). In the standard medium, the phases at 1, 5, and 15 days, were quite similar and the cell population was mainly in the early apoptotic phase. A similar dead cell distribution was found at 1 and 5 days in the osteogenic medium. However, the number of cells in the early apoptotic phase at 5 days was significantly higher than in the standard medium (*p* < 0.05), or than at 15 days in the osteogenic medium (*p* < 0.005), where the predominant dead cells were those in late apoptosis (*p* < 0.05 compared to the situation after 5 days), and necrosis (*p* < 0.01 compared to the situation after 5 days). Furthermore, at 15 days both necrotic and late apoptotic cells were significantly higher than early apoptotic cells and the necrotic population was significantly higher than the late apoptotic population (*p* < 0.001). These results are consistent with the findings on DAPI staining and TUNEL assay. In short, under osteogenic conditions, the various phases of the cell death process seem to have been completed in the time window considered, with a transition from early to late apoptosis/necrosis.

#### Activation of apoptogenic gene expression

Activation of the apoptotic process was examined by measuring *BAX* and *BCL2* gene expression using qRT/PCR. While HK-2 cells grown under standard conditions expressed more* BCL2* gene after 15 days than on days 1 or 5 (*p* < 0.05), those grown in the osteogenic medium had a gradually statistically significant upregulated *BAX* expression compared with *BCL2* (*p* < 0.05 compared with days 1 or 5) (Fig. 3c).

#### Caspase activation

Since the onset of apoptosis is characterized by caspase activation in the cytosol, the levels of cleaved caspase-9 (an initiator caspase), and cleaved caspase-3, -6, -7 (effector caspases) and cleaved poly (ADP-ribose) polymerase (PARP) were measured using In-Cell Western analysis (Fig. 3d). Cells grown in standard conditions showed no difference in cleaved caspase-9 levels over time, while cleaved caspase-3 (*p* < 0.001) and-6 (*p* < 0.01) were significantly upregulated at 15 days compared with their levels at 1 and 5 days, and there was no difference in cleaved caspase-7. In the HK-2 cells grown in the osteogenic medium, on the other hand, there was a significant upregulation of cleaved caspase-9 (*p* < 0.05) and -3 (*p* < 0.0001) at 15 days, compared with the situation after 1 and 5 days, or with cleaved caspase-9 and -3 levels at 15 days in standard conditions (*p* < 0.05), while caspase-6, and -7 remained unchanged.

In osteogenic conditions, cleaved PARP was significantly upregulated after 15 days, by comparison with its level after 1 and 5 days (*p* < 0.0005), or after 15 days in standard medium (*p* < 0.05) (Fig. 3d).

Taken together, these results indicate that the HK-2 cells in osteogenic medium underwent apoptosis via sequential caspase activation, and only revealed dead cells near or in the nodules containing calcium phosphate deposits, suggesting that the presence of apoptotic cells in the nodules was associated with the calcification process.

### Osteogenic transdifferentiation of HK-2 cells

**Fig. 4:**
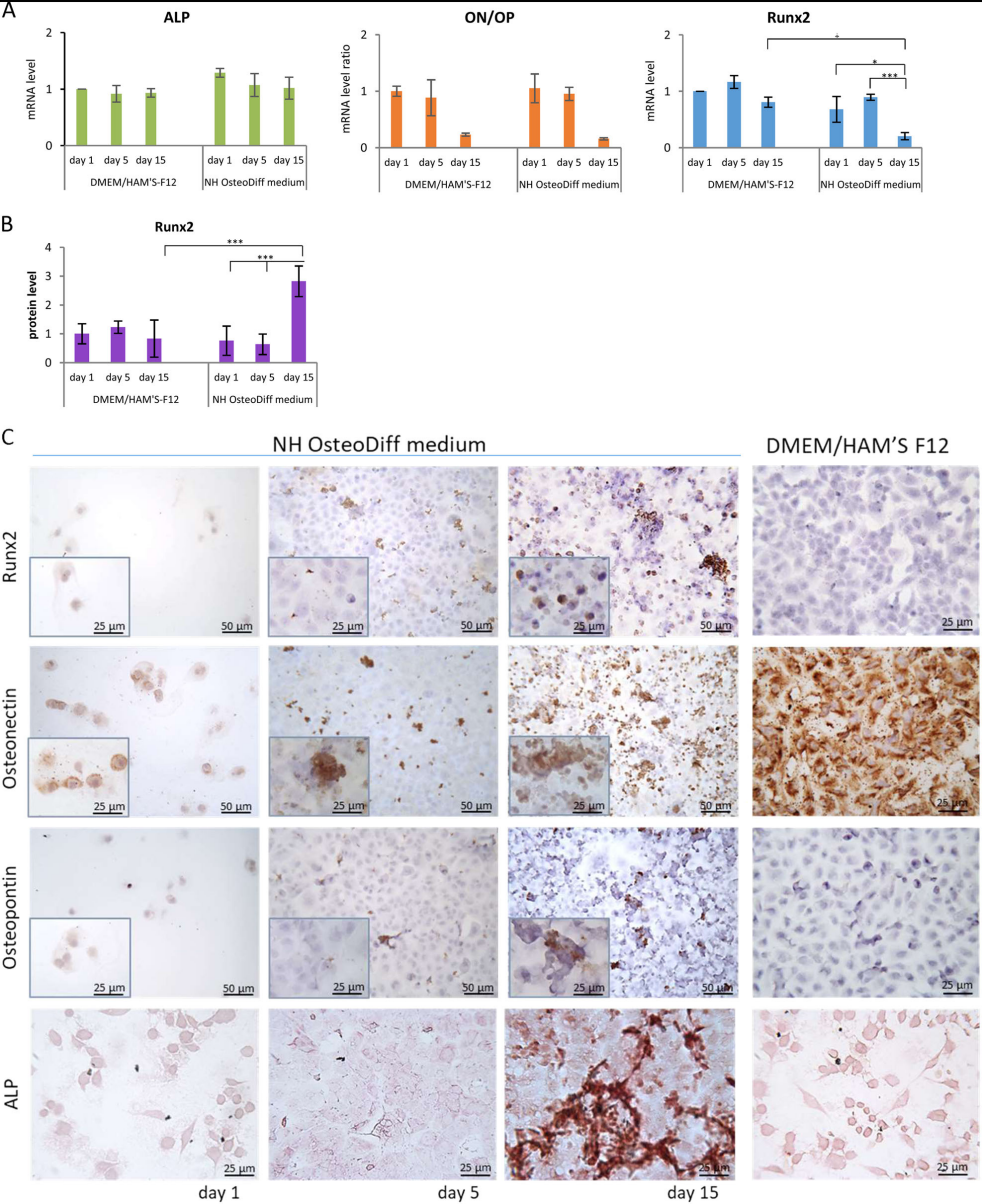
An osteogenic-like process is activated in HK-2 cells grown in osteogenic conditions. **a** qRT/PCR of osteogenesis-related genes: *Runx2*, *ALP*, osteonectin (*ON*) and osteopontin (*OP*). For ON and OP, the results are expressed as the ratio of ON to OP, indicating the balance between pro- and anti-osteogenic factors. Data are presented as the mean ± SD of three separate experiments. *p < 0.05; ***p < 0.005. **b** Runx2 protein quantification by in-cell Western assay. Data are presented as the mean ± SD of three separate experiments. ***p < .0.005. **c** Immunolabeling of Runx2, osteonectin and osteopontin, and ALP activity in HK-2 cells in osteogenic and standard conditions. Immunostaining images of cells in NH OsteoDiff medium: Bar = 50 µm. Inserts show details of immunostaining. Bar = 25 µm. Immunostaining images of cells in DMEM/HAM'S F12: Bar= 25 μm. ALP images: Bar = 25 µm

The expression of osteogenesis-related genes like Runx2, ALP (early osteogenic programming genes), osteopontin (OP) and osteonectin (ON) (later osteogenic programming genes), was measured using qRT/PCR over the course of osteogenic induction (at 1, 5, and 15 days) to see if calcium phosphate deposition was related to an osteogenic-like process. No differences emerged in the *ALP* or *ON/OP* expression of HK-2 cells grown in standard versus osteogenic medium (results are given as the ratio of ON to OP, indicating the balance between pro- and anti-osteogenic factors; Fig. 4a). *Runx2* expression was significantly lower after 15 days in the osteogenic medium than at 1 and 5 days or at 15 days in the standard medium (*p* < 0.05), but using In-Cell Western to measure Runx2 at protein level showed an upregulation at 15 days, by comparison with its level after 1 and 5 days, or at 15 days in standard medium (*p* < 0.005) (Fig. 4b). Immunolabeling with Runx2 confirmed these results, and showed in osteogenic conditions that Runx2 expression at 15 days mainly involved circular cells, primarily in nuclear position, and occurred around nodules (Fig. 4c).

Although *ALP* mRNA levels did not change with time or type of growth medium, we detected ALP activity at 15 days under osteogenic conditions (Fig. 4c).

Assessing ON and OP expression at protein level revealed staining differences between 1, 5, and 15 days in the cells grown in osteogenic medium (Fig. 4c). At 1 day ON staining was present mainly localized around the nucleus. At 5 days, ON staining was strong in what looked like calcium phosphate deposits, but weak and diffuse in the cytoplasm of almost all cuboidal cells forming the monolayer. At 15 days, it was strong in circular cells, nodules and cell fragments (possibly matrix vesicles). There was almost no OP staining at 1 and 5 days, and it only involved a few nodules at 15 days. OP staining appeared to be less intense and more confined than ON (Fig. 4c). In standard condition, we found cells ON stained too, but no OP, Runx2 and ALP staining was present (Fig. 4c).

These results suggest that an osteogenic process was underway at 15 days, and might have persisted afterwards.

## Discussion

Crystal binding to renal tubular cells may be the cause underlying nephrocalcinosis and nephrolithiasis, and healthy cells may develop a crystal-binding phenotype under certain pathological circumstances. We have already reported that GDNF-silenced HK-2 cells grown in osteogenic conditions for 15 days triggered calcium phosphate deposits, providing evidence of caspase-independent cell death prompting this calcification process. We have also found calcium phosphate aggregates (albeit in much smaller amounts) in wild-type (WT) HK-2 cells cultured in osteogenic conditions^27^.

The present study aimed to replicate the previous experiments in WT HK-2 cells and to investigate the relationship between mineralization and apoptosis and/or osteogenesis in human renal proximal tubular epithelial cells grown in osteogenic medium. We successfully induced mineralization/calcification of HK-2 grown in osteogenic medium, with Ca and P deposition increasing overtime. These calcified cells exhibited characteristic signs of caspase-dependent apoptosis. We also found that calcifying HK-2 cells displayed osteogenic markers, such as Runx2, ALP, ON and OP. Monitoring the cells for 15 days showed that apoptosis started within 5 days of induction, when the first small Ca and P crystals became apparent.

Characteristic cellular and nuclear signs of apoptotic induction, such as cell shrinkage, nuclear condensation, and chromatin marginalization and fragmentation, were seen after 5 days. Cells in early apoptosis (as determined using annexin V-FITC assay) were prominent at this stage of apoptotic induction. Annexin V-FITC is a fluorescent probe that binds to phosphatidylserine (PS) in the presence of calcium. At the onset of apoptosis, PS (normally found on the inside of the plasma membrane) translocated to the outside of the membrane due to the loss of membrane phospholipid asymmetry, and thus becomes available to annexin V. PS is an acidic phospholipid of the cell membrane and a key component of the lipid–calcium–phosphate complexes that initiate mineral deposition during bone formation^43,44^. It has a high affinity for calcium^45^, so apoptotic bodies may accumulate Ca and P on their outer membrane surface through their external PS. This hypothesis is supported by the finding that apoptotic bodies isolated from vascular smooth muscle cells (VSMC) accumulate radiolabeled calcium from the incubation solution^31^, making the initial formation and growth of the mineral phase on the outer membrane surface of apoptotic bodies quite likely. HK-2 cells might have developed active Ca and P crystal nucleation by day 5, when apoptotic cells retained their plasma membrane integrity, but PS had translocated over the cell membrane.

We were unable to obtain information on the composition of the commercially available osteogenic medium used (NH OsteoDiff medium, purchased from Miltenyi Biotec), but can safely assume that it contains Ca and P. Primary calciprotein particles (CPP) containing amorphous Ca and P form in artificial fluids when phosphate and calcium are added. With time, these primary CPP spontaneously turn into secondary CPP, which contain crystalline calcium phosphate^46,47^. Aghagolzadeh et al.^48^ generated CPP by adding a certain amount of calcium and inorganic phosphate to phenol-free DMEM with 10% of FBS. They found that storing the medium at 37 °C generated primary and secondary CPP after 1 and 7 days, respectively. The Authors demonstrated that exposing VSMC to secondary CPP led to a pronounced and consistent accumulation of cell-bound calcium. The extent of CPP-induced VSMC calcification depended on CPP concentration. CPP also led to early apoptosis, concomitant with the calcium accumulation. A similar mechanism can be hypothesized for our HK-2 cells exposed to commercial osteogenic medium. CPP might form during incubation of the culture at 37 °C and, after 5 days, with their deposition around cells occurring in the early apoptotic stage.

Calcium oxalate, calcium phosphate and other crystals are known to be capable of inducing cell death, especially in renal proximal tubule cells^14^, possibly depending on crystal size. Sun et al.^49^ demonstrated that nano-sized crystals primarily caused apoptosis, while micron-sized crystals caused necrosis. Lysosomes may internalize nano-sized crystals, suffering damage capable of initiating apoptosis as a result. Nano-sized crystals can also pass through pores into the nucleus, prompting DNA cleavage into regular fragments, an important characteristic of apoptotic cell death. A variety of crystals enter cells by phagocytosis, inducing a caspase-independent cell death called necroptosis^50^.

In our experimental setting, both late apoptotic and necrotic cells were prominent at 15 days, with abundant Ca and P deposition confined to multicellular aggregates or nodules, where cells in late apoptosis were visualized by TUNEL enzymatic labeling and DAPI co-staining assay. At 15 days, there was also caspase -9 and -3 activation, and PARP degradation, indicating a caspase-dependent cell death process. Caspase activation during apoptosis results in the cleavage of critical substrates, including PARP, thereby precipitating the dramatic morphological changes of apoptosis^51,52^.

The importance of apoptosis in the HK-2 cell calcification process was underscored by our findings concerning *BCL2* and *BAX* gene expression. In osteogenic medium, *BCL2* was less expressed than *BAX* as of day 1.The Bcl-2 family of proteins has an important regulatory role in apoptosis, as an activator (BAX), or inhibitor (Bcl-2)^53^, and the ratio of BAX to Bcl-2 has a key role in regulating the apoptotic process^54,55^. The higher BAX/Bcl-2 ratio in the osteogenic than in the standard medium as of day 1 strongly supports the hypothesis that apoptosis triggers the HK-2 calcification process preceding calcium phosphate crystal deposition. In fact, no calcium phosphate was visible on von Kossa staining on day 1. Calcified deposits appeared by day 5, but only in or near areas where cells were apoptotic, suggesting that early calcification is linked to HK-2 cell death, and pointing to apoptotic areas serving as a nidus for this process.

Unwanted calcification (as in ectopic calcification and nephrolithiasis) was long considered a passive, physical and chemical sign of an irreversible degenerative process^56^. Accumulating evidence, particularly in vascular calcification, now suggests that calcium and phosphate precipitation is the outcome of a complex series of events that lead to an active, tightly-regulated process resembling bone mineralization, with VSMC transition towards a chondrogenic/osteogenic phenotype, resulting in mineralization^57^. Thus, we cannot exclude that cell-bound crystals may trigger intracellular transduction pathways leading to the possibility of ectopic renal calcification being an osteogenic‐like process.

After 5 days of osteogenic induction, we saw ON immunolabeling at the sites of crystal deposition. ON is a calcium-binding glycoprotein secreted by osteoblasts during bone formation to initiate mineralization and promote crystal formation. ON also shows an affinity for collagen, and binds collagen and hydroxyapatite in separate domains. Intriguingly, the HK-2 cells in our standard culture conditions and after 1 day in osteogenic medium were ON immunolabeled too. This is hardly surprising, since ON has been implicated in several other biological functions too, such as modulating cell proliferation, and promoting cell attachment and spreading^58^. Thus, ON constitutively secreted by HK-2 cells might have promoted the calcification process in osteogenic medium at 5 days.

On the other hand, OP was not detected in the HK-2 cells grown in standard conditions, nor in those grown for 5 days in osteogenic medium. It did appear in some nodules in osteogenic medium after 15 days, though immunolabeling was less intense than for ON. OP belongs to a family of secreted acidic proteins with an abundance of negatively charged amino acids. Thanks to an overall negative charge, specific acidic motifs, the fact that OP is intrinsically disordered, allowing for open and flexible structures, OP can bind strongly to calcium atoms available on the surfaces of various biomineral crystals^59^, inhibiting mineralization and thereby regulating crystal growth^60^. Overall, our findings concerning ON and OP immunolabeling suggest that a cell-mediated pro-calcifying process is taking place.

Runx2 and ALP were upregulated at 15 days, pointing to an osteogenic-like process underway. We cannot say when this process started, or precisely how it developed, because it was not monitored in the time window between 5 and 15 days. It was clear, however, that Runx2 (considered the master gene of osteoblast differentiation) became upregulated after calcification began, unexpectedly suggesting that osteogenesis may not be the primary pathogenic event in the onset of cell calcification. The same situation was very recently described in the vascular calcification of uremic rats fed a high phosphate diet^61^: examining the effects of 5/6 nephrectomy and a high phosphate diet on aortic gene and protein expressions at different time points after nephrectomy, the Authors found that bone morphogenic protein 2 and Runx2 expression developed after the onset of calcification. The Authors concluded that the osteogenic phenotype in vascular calcification might be a consequence of calcium deposition rather than a cause. This could be true of calcifying HK-2 cells too.

In conclusion, we showed that HK-2 cell mineralization is induced by osteogenic culture conditions, and coincides with areas where apoptosis occurs, suggesting a sequence of events. In osteogenic conditions, cells promptly underwent apoptosis, and the subsequent release of apoptotic bodies allowed mineral ions and/or CPP present in the medium to accumulate. HK-2 cells grown in standard conditions secreted ON, and at this stage ON seemed to contribute to biomineral crystal formation. This phase was followed by an osteogenic-like process with Runx2, ALP and ON upregulation, suggesting that the osteogenic phenotype is a consequence rather than a cause of calcium phosphate deposition.

This study further corroborates the importance of cell death in cell calcification. Taken together, the present data point to a process very similar to vascular calcification having a role in the onset of human nephrocalcinosis/nephrolithiasis.

## Materials and methods

### Cell culture

The HK-2, immortalized human renal proximal tubular epithelial cell line, derived from normal adult human kidney, was purchased from American Type Culture Collection (ATCC) (CRL-2190TM). HK-2 cells were maintained in a mixture of HAM’S F12 and Dulbecco’s modified Eagle’s growth medium (DMEM/HAM’S-F12; EuroClone, CelBio) supplemented with 10% heat-inactivated FBS (HI-FBS), 2 mM l-glutamine, 100 U/ml penicillin, and 100 μg/ml streptomycin (EuroClone, CelBio). Cells were grown in a humidified atmosphere of 5% CO_2_ and 95% air at 37 °C. The cells were seeded at an appropriate cell density for different assays and left to grow to 80% confluence, then synchronized routinely by incubating cells in serum-free medium for 24 h prior to each experiment. The cells were exposed to different experimental conditions. Each experiment was performed at least three times.

#### Osteogenic culture of HK-2 cells

HK-2 cells were cultured on six-well tissue culture plates (Falcon™ PolystyreneMicroplates, Thermo Scientific) at a density of 4.5 × 10^4^ cells per well in commercially-available osteogenic medium (NH OsteoDiff medium, Miltenyi Biotec) for 1, 5, or 15 days. Control conditions were established by culturing cells in DMEM/HAM’S-F12 medium supplemented with 10% HI-FBS and pen/strep (standard medium). The osteogenic and standard media were both replaced every 2–3 days for up to 15 days.

#### Cell growth and viability assessment

Cells were plated at 10 × 10^3^ cells/well on 24-well tissue culture plates (Falcon), and grown to 50% confluence in culture medium, then the medium was switched to 1% FBS 24 h before the experiments to induce quiescence. A standard or osteogenic medium was added to the cells and changed every 2 days. Proliferation was assessed at different times (on days 0, 1, 2, 3, 4, and 7), by means of cell counts and colorimetric assays^62,63^. Briefly, cells were fixed with methanol for 10 min, then stained with 1% methyl blue in 0.01 M borate buffer (pH 8.5) for 30 min. After repeated washing, the unbound staining solution was eluted with a 1:1 mixture of ethanol and 0.1 N HCl, and read at an absorbance of 650 nm. Methyl blue only stains cells attached to the substrate before fixation (i.e. living cells), and thus quantitates their proliferation and viability.

### Detecting and quantitating calcification

#### von Kossa staining

von Kossa staining was used to detect calcium crystal deposition. Cells were seeded in an eight-well chamber slide system (4×10^3^ cells/well; Nunc Lab-Tek Chamber Slide system; eight wells on Permanox; Thermo Scientific), incubated in standard or osteogenic media for 1, 5, or 15 days, then washed twice with PBS. They were then fixed in PBS-formalin for 10 min. After washing twice with PBS and once with water, a 2% silver nitrate solution was added. The slides were exposed to UV light for 30 min and after rinsing once again with water, sodium thiosulfate (5%) was added for 3 min. The slides were again rinsed in water, then hematoxylin was added for 5 min to counterstain the nuclei. After rinsing in water for the last time, the slides were mounted in glycerol and water solution, and visualized using a Diaplan light microscope (Leitz). To quantitate calcium deposition, images were acquired using a Micropublisher 5.0 RTV Camera (Q Imaging), and morphometric analysis of the von Kossa staining was performed.

#### Environmental SEM (ESEM) analysis

After seeding on eight-well slides (4 × 10^3^ cells/well; Nunc) and treatment, cells were washed twice with PBS and fixed in methanol for 10 min. To assess the chemical composition of the cell nodules, environmental SEM (ESEM) analysis with X-ray fluorescence, coupled with energy-dispersive spectroscopy (XRF-EDS), was performed directly on the cells grown on the plastic slides using an ELEMENT instrument (EDAX). This method enables the identification of inorganic compounds within a biological matrix typically comprising carbon, oxygen, and hydrogen. The spectra gathered in the X-ray fluorescence show the peaks of all the elements involved, so a semiquantitative measure of the composition of the inclusions can be obtained by analyzing the net intensities calculated by the peak integral with background line subtraction.

### Detecting and quantitating cell death

#### Cell morphology analysis

The cells were seeded on eight-well chamber slides (4 × 10^3^ cells/well; Nunc), cultured for 1, 5, or 15 days in normal and osteogenic media, then washed with PBS and fixed in cold methanol for 10 min at RT. Changes in cell morphology were examined first under an inverted light-phase contrast microscope (EVOS XL Core Cell Imaging System), that enabled a rapid analysis of cell culture status. Then, to obtain more morphological details, cells were observed under the differential interference contrast (DIC) microscope (DMI600CS-TCS SP8; Leica Microsystems), and analyzed with LAS AF software (Leica Microsystems). The DIC optics create a virtual relief image, enabling the morphological analysis of transparent objects. Images were acquired using a DFC365FX camera (Leica Microsystems).

#### Detecting in situ cell death with the TUNEL and DAPI co-staining assay

DNA fragmentation was assessed using the terminal deoxynucleotidyl transferase dUTP nick-end labeling (TUNEL) assay (TUNEL In Situ Cell Death Detection Kit, Roche). Cells were seeded on eight-well chamber slides (4 × 10^3^ cells/well; Nunc), and cultured for 1, 5, or 15 days in standard and osteogenic media. Cell cultures were fixed with 4% formaldehyde in PBS for 10 min.at RT, and permeabilized with 0.1% (vol/vol) Triton X-100 in aqueous 0.1% (wt/vol) sodium citrate for 2 min on ice. Then they were incubated for 1 h at 37 °C with a TUNEL reaction mixture (comprising a nucleotide mixture in reaction buffer and TdT) and subsequently stained with DAPI (1 µg/ml) for 10 min to detect fragmented and condensed nuclei. The slides were washed three times with PBS, mounted in glycerol and water solution, examined under the DIC microscope (DMI600CS-TCS SP8; Leica Microsystems), and analyzed with the LAS AF software (Leica Microsystems). Images were acquired using a DFC365FX camera (Leica Microsystems). Cells treated with 20.0 U/μl DNase I for 20 min were used as a positive control.

#### Simultaneous annexin V-FITC and propidium iodide staining

Cell apoptosis was quantified by flow cytometry at the different time points, measuring annexin V and propidium iodide (PI), and using a kit from Affymetrix-eBioscience according to the manufacturer’s instructions. Briefly, after washing with PBS, the cells were detached using trypsin and resuspended at a density of 200-500 × 10^3^ cells/ml in 100 μl annexin-binding buffer (10 mM HEPES, pH 7.4; 140 mM NaCl, and 2.5 mM CaCl_2_) containing 5 μl of annexin V-FITC. This mixture was incubated for 10 min. in the dark at RT, then the cells were washed with binding buffer and resuspended in the same buffer containing PI. At least 1 × 10^4^ cells were analyzed, and apoptotic stages were examined by flow cytometry using a CytoFLEX cytometer (Beckman Coulter). AnnexinV-positive/PI-negative and annexinV-positive/PI-positive cells were considered as being in the early and late phases of apoptosis, respectively. AnnexinV-negative/PI-positive cells were considered necrotic. All cell populations were counted together and defined as the total dead cell population. Staurosporine (1.0 μm)-treated cells were used as a positive control.

#### Caspase activation

Caspase activation was measured using In-Cell Western analysis, as described elsewhere^27^. Briefly, cells were seeded on a 96-well plate (2 × 10^3^ cells/well; Nunc) and cultured in standard or osteogenic media for 1, 5, or 15 days. They were then fixed in cold methanol for 10 min.at RT, and washed five times with 0.1% Triton X-100 in PBS. The samples were blocked in a solution of 5% milk in PBS containing 0.1% Triton X-100 for 40 min.at RT with moderate shaking, followed by incubation with specific primary antibodies (Table 1) overnight at 4 °C in a humidified chamber. β-tubulin served as an internal control. The plates were washed five times with 0.1% Triton X-100 in PBS and gently agitated for 5 min at RT. Secondary antibody (IRDye 800CW donkey anti-rabbit, 1:800, from LI-COR, Biotechnology, Lincoln, NE, USA) was added to each well and incubated in the dark for 60 min at RT with gentle shaking. Finally, the plates were scanned at 800 nm and the intensity of the labeled proteins was measured using the Odyssey Infrared Imaging System (LI-COR). Negative controls were obtained by omitting the primary antibody during the incubation steps, and background values were obtained by omitting primary and secondary antibodies. The data are shown as the mean ± SD. Staurosporine (1.0 μm)-treated cells were used as a positive control.

**Table 1:** Primary antibodies and dilutions used for In-Cell Western assays

Target	Clone	Host	Manufacturer	Code	Dilution
Cleaved Caspase-9 (Asp330)	D2D4	rabbit	Cell Signaling Technology	CST-7237	1:1000
Cleaved Caspase-3 (Asp175)	5A1E	rabbit	Cell Signaling Technology	CST-9664	1:1000
Cleaved Caspase-6 (Asp162)	—	rabbit	Cell Signaling Technology	CST-9761	1:1000
Cleaved Caspase-7 (Asp198)	D6H1	rabbit	Cell Signaling Technology	CST-9761	1:1000
Cleaved PARP (Asp214)	D64E10	rabbit	Cell Signaling Technology	CST-5625	1:1000
Runx2/CBFA1	—	rabbit	Novus Biologicals	NBP1-77461	1:50
β-Tubulin	H235	rabbit	Santa Cruz Biotechnology	sc-9104	1:50

### Cell osteogenic differentiation

#### Alkaline phosphatase staining

Cytochemical staining for alkaline phosphatase (ALP), an enzyme involved in bone matrix mineralization and an early marker of committed osteogenic cells, was done with a commercially available kit (Leukocyte Alkaline Phosphatase (LAP) kit, Sigma-Aldrich). Cells were seeded on eight-well chamber slides (4 × 10^3^ cells/well; Nunc). After incubation in standard or osteogenic media for 1, 5, or 15 days, cells were washed twice with PBS, then fixed with pre-chilled methanol for 10 min at RT. After removing the methanol, cells were then incubated in a mixture of naphthol AS-BI alkaline solution with fast red violet LB, according to the manufacturer’s instructions. The resulting insoluble diffuse, red dye deposit indicates sites of alkaline phosphatase activity. Slides were mounted in a glycerol and water solution, and analyzed under a Leica DMIL LED phase-contrast inverted microscope (Leica Microsystems). Images were acquired using a LEICA ICC50W camera.

#### Immunolabeling with osteogenic markers

HK-2 were cultivated on eight-well chamber slides (4 × 10^3^ cells/well; Nunc) in standard or osteogenic medium for 1, 5, or 15 days. Cells were washed twice with PBS and fixed with cold methanol for 5 min at RT. After methanol removal, the specimens were treated with 2% H_2_O_2_ in PBS (pH 7.4) for 15 min at RT to block endogenous peroxidase activity, then incubated with 2% normal goat serum (Sigma-Aldrich) for 30 min at RT to prevent non-specific antibody binding.

Cells were than incubated overnight at 4 °C in a humidified chamber with a mouse Runx2 monoclonal antibody (MO5, clone 1D2, Abnova) at the final concentration of 12 µg/ml in PBS, or with a rabbit osteonectin (ON) polyclonal antibody (Millipore) at 1:800 dilution in PBS, or a rabbit osteopontin (OP) polyclonal antibody (Chemicon) at 1:4000 dilution in PBS. Samples were then treated with HRP-linked goat anti-mouse antibody (dilution 1:700) to highlight Runx2, or with DakoCytomation EnVision + System-HRP Labeled Polymer anti-rabbit (DAKO Corporation) to reveal ON and OP. The chamber slides were incubated in a humidified chamber at RT for 30 min. Signals were visualized using the chromogen 3,3-diaminobenzidine-tetrachloride (DAB, DAKO), and cells were counterstained with hematoxylin. The specificity of the immunolabeling was confirmed in treated cells without the primary antibody, or with nonimmune rabbit IgG (Sigma-Aldrich). Slides were analyzed under the Diaplan light microscope (Leitz), and images were acquired using a Micropublisher 5.0 RTV camera (Q Imaging).

### Quantitative real-time PCR (qRT/PCR)

Total RNA was extracted from cell cultures at 1, 5, or 15 days using the RNeasy Mini Kit (Qiagen Limburg, NL) according to the manufacturer’s instructions, and following the spin column protocol. RNA quantity and quality were assessed by spectrophotometric analysis using a NanoDropND-1000 (Thermo Fisher Scientific, Waltham, MA, USA), and by capillary electrophoresis using an Agilent 2100 Bioanalyzer (Agilent Technologies, Santa Clara, CA, USA). RNA samples with an A260/A280 ratio between 1.8 and 2, and an RNA integrity number (RIN) of at least 9 were used for qRT/PCR. A total amount of 100 ng of total RNA was reverse-transcribed in a final volume of 20 μl containing 5 mM MgCl_2_, 1 mM dNTPs, 2.5 μm random hexamers (Applied Biosystems), 1 U/μl RNAse inhibitor (Applied Biosystems), and 2.5 U/μl MuLV reverse transcriptase (Thermo Fisher Scientific) in a buffer comprising 50 mM KCl and 10 mM Tris/HCl (pH 8.3). Reactions were performed on a 2720 Thermal Cycler (Thermo Fisher Scientific) using the following thermal profile: RT for 10 min, 42 °C for 30 min, 65 °C for 5 min, and 4 °C for 5 min. The primers used are listed in Table 2. Primer pairs for the region of interest were designed using Primer3 software ver.4.0 (http://primer3.ut.ee), adopting stringent parameters to ensure successful amplification and a convenient experimental design. The National Center for Biotechnology Information (NCBI) Primer-BLAST program was used for in silico specificity analysis (www.ncbi.nlm.nih.gov/tools/primer-blast/index.cgi), after which each primer pair was validated. Microchip electrophoresis on an Agilent 2100 Bioanalyzer, Sanger sequencing, and melting curve analyses were used to measure the specificity of the PCRs. Amplification curves were established for all the primers and showed an efficiency of 85%, at least. qRT/PCR was performed using an iCycler Thermal Cycler (Bio-Rad, Hercules, CA, USA) and SYBR Green I technology with the iQTM SYBR Green Master Mix (Bio-Rad) in a final volume of 20 μl containing 1 μl of reverse-transcribed cDNA template. An appropriate primer concentration (0.3 μm) was used, and the annealing temperatures are listed in Table 1. Data were analyzed using the ΔΔCt method, normalizing the data to two different housekeeping genes (glyceraldehyde 3-phosphate dehydrogenase (*GAPDH*) and hypoxanthine guanine phosphoribosyl transferase (*HPRT1*) according to the guidelines on the minimum information for the publication of quantitative real-time PCR experiments (MIQE)^64^. The normalized relative quantitation (nRQ) was calculated as 2^−ΔΔCt^. A melting curve analysis was performed to identify any non-specific amplification products. All results were obtained from three separate experiments performed in triplicate.

**Table 2:** Primer sequences used in the qRT/PCR analyses

Primers	Nucleotide sequence (5′–3′)	T annealing (°C)	NCBI reference sequence	Efficiency (%)
GAPDH Fw GAPDH Rev	GAAGGTGAAGGTCGGAGT TGGCAACAATATCCACTTTACCA	60	NM_17851.1	99.0
HPRT1 Fw HPRT1 Rev	CCTGGCGTCGTCATTAGTGA TCTCGAGCAAGACGTTCAGT	60	NM_000194.2	97.8
Osteonectin Fw Osteonectin Rev	CCTGGATCTTCTTTCTCCTTTGC ATCAGGCAGGGCTTCTTGCT	60	NM_001309443.1	97.9
Osteopontin Fw Osteopontin Rev	CGAGACCTGACATCCAGTACC GATGGCCTTGTATGCACCATTC	62	NM_001251830.1	96.2
Runx2 Fw Runx2 Rev	CATTTCAGATGATGACACTGCC GGATGAAATGCTTGGGAACTG	62	NM_001024630.3	95.2
ALP Fw ALP Rev	GGCAACTCTATCTTTGGTCTG GTTGTTGTGAGCATAGTCCA	60	NM_000478.4	95.0
BAX Fw BAX Rev	GCCGTGGACACAGACTCC AAGTAGAAAAGGGCGAAACC	60	NM_001291428.1	91.2
BCL-2 Fw BCL-2 Rev	TCATGTGTGTGGAGAGCGTCAA CAGCCAGGAGAAATCAAACAGAGG	60	NM_000633.2	88.9

### Morphometric analysis

Morphometric analysis was done with ImagePro Plus software (Media Cybernetics). For each experimental sample, up to 15 images at 200X or 400X magnification were analyzed. Signals were acquired for all the images with the same brightness and contrast characteristics from three different slides, and quantities were expressed as the percentage of the mean area covered by pixels^27^.

### Statistical analysis

Data are presented as mean ± SD. Multiple group means were compared using ANOVA with a between-within design and Bonferroni’s correction. Two group means were compared using *T*-test. Data from the morphometric analysis were examined using a non-parametric test (the Mann–Whitney *U* test), and statistical significance was established with the Primer software (McGraw-Hill). A *p*-value of less than 0.05 was considered statistically significant.

## Electronic supplementary material

Author Contribution form
