# Supplementary material for: Human proximal tubular cells can form calcium phosphate deposits in osteogenic culture: role of cell death and osteoblast-like transdifferentiation
Source: Cell Death Discov. 2019 Jan 28;5:57. doi: 10.1038/s41420-019-0138-x (PMC6349935; doi:10.1038/s41420-019-0138-x)
Supplement: Supplementary file 1 — Author Contribution form [file 41420_2019_138_MOESM1_ESM.pdf]

DECLARATION OF CONTRIBUTIONS TO ARTICLE

ADMC

Manuscript Number: CDDIS-18-2987

Journal Name: *Cell Death & Disease* (the 'Journal')

Proposed Title of the Contribution:

HUMAN PROXIMAL TUBULAR CELLS CAN FORM CALCIUM PHOSPHATE DEPOSITS IN OSTEOGENIC CULTURE: ROLE OF CELL DEATH AND OSTEOBLAST-LIKE TRANSDIFFERENTIATION (the 'Contribution)

Author(s):

Giovanna Priante, Monica Ceol, Lisa Gianesello, Claudio Furlan, Dorella Del Prete, Franca Anglani (the 'Authors')

For all *CDDis* articles, each person named as an author in the published version must be able to show he or she has contributed substantially to the article.

Authorship credit should be based on 1) substantial contributions to conception and design, acquisition of data, or analysis and interpretation of data; 2) drafting the article or revising it critically for important intellectual content; and 3) final approval of the version to be published. Authors should meet conditions 1, 2 and 3.

Any person who cannot be shown to have made a substantial contribution to the article cannot be listed as an author in the final version. The name of any person who is deemed to have made a minor contribution can, however, appear in the Acknowledgments section of the article.

Please complete the table below to indicate the contributions of all named authors to the manuscript.

Author Full Name:	Specification of Contribution to the Manuscript:
Giovanna Priante	Planned and conducted the experiments, analyzed the data and wrote the manuscript
Monica Ceol	Conducted the experiments, analyzed the data
Lisa Gianesello	Conducted the experiments, analyzed the data
Claudio Furlan	Performed ESEM analyses
Dorella Del Prete	Analyzed the data and revised the manuscript
Franca Anglani	Co-ordinated and planned the experiments and wrote the manuscript

Please complete the table below to indicate the contributions of all named authors to the figures.

Figure 1:

GP generated the data and prepared panels A and B; GP assembled the figure

Figure 2:

GP generated the data and prepared panels A and B; CF generated ESEM data and images and GP prepared panels C, D and E; GP assembled the figure

Figure 3:

LG generated images and GP generated the data and prepared panels A, B, C and D; GP assembled the figure

Figure 4:

GP generated the data and prepared panels A and B; GP and LG generated the data and prepared panel B; MC generated the immuno histochemistry data and images; GP prepared panels C; GP generated cytochemical staining for ALP and data panel D; GP assembled the figure

Figure 5:

Figure 6:

Signed for and on behalf of the Author(s):	Print Name:	Date:
